# Hydrogen Sulfide Donor NaHS Reduces Organ Injury in a Rat Model of Pneumococcal Pneumosepsis, Associated with Improved Bio-Energetic Status

**DOI:** 10.1371/journal.pone.0063497

**Published:** 2013-05-23

**Authors:** Hamid Aslami, Wilco P. Pulskens, Maria T. Kuipers, Aafkeline P. Bos, André B. P. van Kuilenburg, Ronald J. A. Wanders, Jeroen Roelofsen, Joris J. T. H. Roelofs, Raphaela P. Kerindongo, Charlotte J. P. Beurskens, Marcus J. Schultz, Wim Kulik, Nina C. Weber, Nicole P. Juffermans

**Affiliations:** 1 Laboratory of Experimental Intensive Care and Anesthesiology (L.E.I.C.A.), Academic Medical Center, Amsterdam, The Netherlands; 2 Laboratory of Genetic Metabolic Diseases, Academic Medical Center, Amsterdam, The Netherlands; 3 Department of Pathology, Academic Medical Center, Amsterdam, The Netherlands; 4 Department of Intensive Care Medicine, Academic Medical Center, Amsterdam, The Netherlands; National Institute of Agronomic Research, France

## Abstract

Sepsis is characterized by a generalized inflammatory response and organ failure, associated with mitochondrial dysfunction. Hydrogen sulfide donor NaHS has anti-inflammatory properties, is able to reduce metabolism and can preserve mitochondrial morphology and function. Rats were challenged with live *Streptococcus pneumonia* or saline and infused with NaHS (36 µmol/kg/h) or vehicle. Lung and kidney injury markers were measured as well as mitochondrial function, viability and biogenesis. Infusion of NaHS reduced heart rate and body temperature, indicative of a hypo–metabolic state. NaHS infusion reduced sepsis–related lung and kidney injury, while host defense remained intact, as reflected by unchanged bacterial outgrowth. The reduction in organ injury was associated with a reversal of a fall in active oxidative phosphorylation with a concomitant decrease in ATP levels and ATP/ADP ratio. Preservation of mitochondrial respiration was associated with increased mitochondrial expression of α–tubulin and protein kinase C–ε, which acts as regulators of respiration. Mitochondrial damage was decreased by NaHS, as suggested by a reduction in mitochondrial DNA leakage in the lung. Also, NaHS treatment was associated with upregulation of peroxisome proliferator-activated receptor–γ coactivator 1α, with a subsequent increase in transcription of mitochondrial respiratory subunits. These findings indicate that NaHS reduces organ injury in pneumosepsis, possibly via preservation of oxidative phosphorylation and thereby ATP synthesis as well as by promoting mitochondrial biogenesis. Further studies on the involvement of mitochondria in sepsis are required.

## Introduction

*Streptococcus pneumoniae* is the leading cause of community acquired pneumonia in patients that require admission to the intensive care unit [1]. Infection with *S. pneumoniae* triggers an intense inflammatory reaction, which can lead to sepsis and multiple organ failure, including acute lung injury and acute kidney injury. Supportive treatment aims to enhance substrate and oxygen delivery to tissues to produce adenosine triphosphate (ATP), thereby preserving the bio–energetic status of organs [2]. However, despite adequate supportive treatment, mortality of sepsis remains high [3].

The mechanisms which underlie sepsis–induced organ injury are complex. The inflammatory response can directly damage mitochondrial DNA, lipids and respiratory complexes, thereby inhibiting oxidative phosphorylation [4] and diminishing ATP availability [2]. In sepsis, mitochondrial dysfunction is associated with adverse outcome [5]. Influencing mitochondrial substrate utilization was shown to improve outcome in sepsis [6]–[8], which may be due to preserved mitochondrial structure and function [9], thereby preserving local ATP levels.

Regulation of cellular metabolism is complex and involves various nuclear transcription factors [10]. Upon stress stimulation, peroxisome proliferator-activated receptor-γ coactivator (PGC)–1α rapidly induces the upregulation of transcription factors resulting in transcription of mitochondrial (mt) DNA, with subsequent formation of several subunits of respiratory complexes inside the mitochondria, including cytochrome c oxidase (COX) 1, NADH1 and NADH6 [10]. Expansion of respiratory complexes increases oxidative phosphorylation and thereby ATP production. ATP availability is regulated by VDAC, a voltage-dependent porin, embedded in the outer membrane of the mitochondria [11]. ATP is transported from the matrix to the cytosol through VDAC in exchange for ADP and phosphate. The function of VDAC is partly regulated by α–tubulin and protein kinase C (PKC)–ε, which can induce closure of VDAC [12], thereby reducing the exchange of ATP with ADP to the cytosol.

Hydrogen sulfide (H_2_S) is an inhibitor of mitochondrial respiration [13] and has been shown to induce a shift in mitochondrial substrate utilization [14], to improve mitochondrial function during ischemic injury [13] and to maintain ATP production under hypoxic conditions [15]. Also, H_2_S has anti–inflammatory properties [16]. In sufficient doses, H_2_S can induce a ‘hibernation–like state’, characterized by hypothermia and low CO_2_ production and O_2_ consumption [17]. Infusion of H_2_S donor NaHS has been shown to reduce lung injury in a rat model of ventilator induced lung injury [18]. Furthermore, a bolus NaHS improved survival when injected simultaneously at the time of induction of sepsis [19] in mice. However, NaHS has a short half life [16]. The effect of extended infusion of high doses of NaHS during sepsis is unknown. Also, the mechanism of the effects of NaHS on mitochondrial function is not known.

In the present study, we hypothesized that NaHS protects against organ damage in a rat model of pneumococcal pneumosepsis by improving bio–energetic status. Furthermore, we studied the pathways by which H_2_S may regulates mitochondrial function.

## Methods

The study was approved by the animal care and use committee of the Academic Medical Centre, Amsterdam, the Netherlands. Animal procedures were carried out in compliance with Institutional Standards for Human Care and Use of Animal Laboratory Animals.

### Induction of Pneumonia

Rats (Sprague Dawley ±350 g, Harlan, The Hague, The Netherlands) were intratracheally inoculated with ∼6×10^6^ CFU of aerosolized *S. pneumoniae* serotype 3 (ATCC 6303; Rockville, MD, USA) using a trans–oral miniature nebulizer under light anesthesia (97% oxygen with 3% isoflurane). Controls received saline. Supplemental fluids were given after inoculation and every 24 hours by intraperitoneal injection of 10 ml/kg of lactated Ringer’s solution.

### H_2_S Donor

Preparations of a H_2_S donor were made fresh on the day of the experiments as described before [18]. NaHS was infused at 36 µmol/kg/h intravenously.

### Experimental Protocol and Groups

Two days after inoculation with *S. pneumoniae* rats were anesthetized with intraperitoneal injection of a mix (0.15 ml/100 g body weight) containing 90 mg/kg ketamine, 0.5 mg/kg medetomidine and 0.05 mg/kg atropine. Anesthesia was maintained by infusion of 50 mg/kg ketamine at 0.5 ml/100 g/hr. Bicarbonate (8.4%) was administered at 1 ml/100 g/hr to maintain normal acid-base balance [20]. A tracheotomy was performed, after which a metal canule was connected to a ventilator (Servo 900C, Siemens, Sweden). Ventilator settings were determined in pilot experiments aiming at normo-pH (7.35–7.45) and tidal volumes of ∼7.5 ml/kg [18]. Detailed ventilatory settings are described in supplementary data. Hemodynamic monitoring was done by a carotid artery catheter connected to a monitor. Urine was collected. After baseline measurements, the animals were randomized to infusion with saline or NaHS, with equal volume load. In the saline control groups, body temperature was maintained at 37°C.

### Exsanguination and Assays

The rats were sacrificed after 4 hours of mechanical ventilation. Lungs were removed *en block*. BALF was obtained by flushing the left lung (3×2.5 ml saline) after ligation of the right lung. Cell counts were determined using a hematocytometer (Z2 Coulter Particle Counter, Beckman Coulter; Florida, USA) and cell differentiation were done on Giemsa-stained cytospins in bronchoalveolar lavage fluid (BALF). Interleukin (IL)–1β, IL–6, TNF–α and CINC3 (R&D Systems; Abingdon, United Kingdom) were measured in BALF and plasma. Alkaline phosphatase (AF) levels were measured by a commercially available kit (Sigma Aldrich, St. Lous, MO) in BALF supernatant using a Hitachi analyzer (Roche BV, Mannheim, Germany). Plasma levels of aspartate amino–transaminase and alanine–transaminase were measured using standard enzymatic methods at 37°C and creatinine by enzymatic PAP (Roche Diagnostics, the Netherlands). To measure protein carbonyls, lung and liver sample (±200 mg) were prepared according to the manufacturers’ instruction (Cayman chemical company, Ann Arbor, Michigan, USA). Glomerular filtration rate was calculated by ([creatinine] urine×urine volume in 24 hours)/[creatinine] plasma.

### Bacterial Outgrowth, Wet Weight and Histopathology

Approximately 0.5 g of lung, liver and spleen tissues were removed, diluted 1∶4 in sterile saline and homogenized. The number of colonizing forming units (CFU) was determined by performing 10-fold dilutions in blood, BALF and organ homogenates on blood agar plates, after 24 hours of incubation at 37°C with 5% CO_2_. The right lung top was Hematoxylin–Eosin (H&E) stained and analyzed by a pathologist who was blinded for group identity. Interstitial inflammation, endothelialitis, bronchitis, edema and pleuritis were scored on a scale of 0–4∶0 for normal lungs, 1 for <25% lung involvement, 2 for 25 − 50% involvement, 3 for 50−75% involvement and 4 for >75% lung involvement. Total histology score is the sum score of all parameters. The remaining right lobes were weighted to determine wet weight, as was the right kidney.

### Mitochondrial Viability Measurements

Part of the liver was removed by modified freeze clamping and placed in liquid nitrogen for ATP and adenosine diphosphate (ADP) measurements and part was placed in isolation buffer. This was done before exsanguination to ensure mitochondrial viability and minimize ATP degradation. Mitochondria were isolated by differential centrifugation steps and respiration was measured polarographically at 37°C using a respiratory system (System S 200A, Strathkelvin Instruments, Glasgow, Scotland). ATP and ADP levels were analysed by HPLC method [21]. The expression of VDAC and its regulators, including α–tubulin and phosphor PKC-ε were measured at protein level in the mitochondrial fraction by western blot. Expression of VDAC and α-tubulin was normalized to expression of prohibitin. Expression of phosphorylated PKCε is shown as a ratio to total PKCε. Expression of PGC1-α, and mtDNA (COX1, NADH1 and NADH6) were measured in liver and lung tissue as well as in plasma and BALF. See methods S1 for more detailed description.

### Statistical Analysis

Data are expressed as mean with SD in the tables and in the figures as mean with SEM. Bacterial outgrowth data was analyzed by Mann-Whitney U test or unpaired t-test according to data distribution. Intergroup differences were analyzed by analysis of variance (ANOVA) and Bonferroni’s post–hoc test, or by a Kruskal Wallis test with Mann−Whitney *U* test dependent on data distribution. A *p* value of <0.05 was considered significant. Statistical analyses were done using Prism (Graphpad Prism 5, CA, USA).

## Results

### *S. pneumoniae* Resulted in Severe Pneumosepsis, Characterized by Lung Injury, Systemic Inflammation, Organ Injury and Low Bio-energetic Status

After inoculation with *S. pneumoniae* but before NaHS infusion, the mortality rate was 11%. These animals were not included. The remaining animals were randomized to NaHS or vehicle infusion. Mean arterial pressure remained above 70 mmHg (table 1). Inoculation resulted in severe macroscopic pneumonia. Hematogenic spread of bacteria was demonstrated by positive cultures of liver or spleen in all animals (figure S1).

**Table 1:** Physiological parameters at baseline and after 4 hours and organ function markers of rats with pneumonia or healthy controls after 4 hours of NaHS infusion or saline.

			Pneumonia		Healthy	
	Time(Hour)		Saline	NaHS	Saline	NaHS
MAP, (mmHg)		T = 0	120±23	121±23	127±20	140±16
		T = 4	91±24	95±19	106±20	101±23
Heart rate, (beats/min)		T = 0	313±61	304±34	306±37	287±37
		T = 4	298±26	143±28*	306±37	136±63^#^
Body temperature, (°C)		T = 0	36.6±1.2	36.6±0.6	37.1±0.8	36.6±0.4
		T = 4	37.4±0.5	24.8±1.6*	36.7±1.3	26.0±1.4^#^
AST, U/L			194±186	121±33	89±44	98±47
ALT, U/L			127±143†	62±26	47±11	49±12
Creatinine, mmol/L			50±9	27±3*	40±7	23±4^#^
GFR, mL/min			1.4±0.4	5.2±2.8*	2.0±1.6	2.4±1.4
Kidney wet weight, g			1.62±0.13†	1.46±0.09*	1.19±0.37	1.38±0.06
Urine protein, mg/mL			0.71±0.58†	0.29±0.39*	0.16±0.25	0.13±0.14

Data are means ± SD.

* Pneumonia+saline vs. pneumonia+NaHS,

#healthy+saline vs. healthy+NaHS,

† pneumonia vs. healthy, p<0.05.

MAP: mean arterial pressure, AST: aspartate aminotransferase, ALT: alanine transaminase, GFR: glomerular filtration rate.

**Figure 1:**
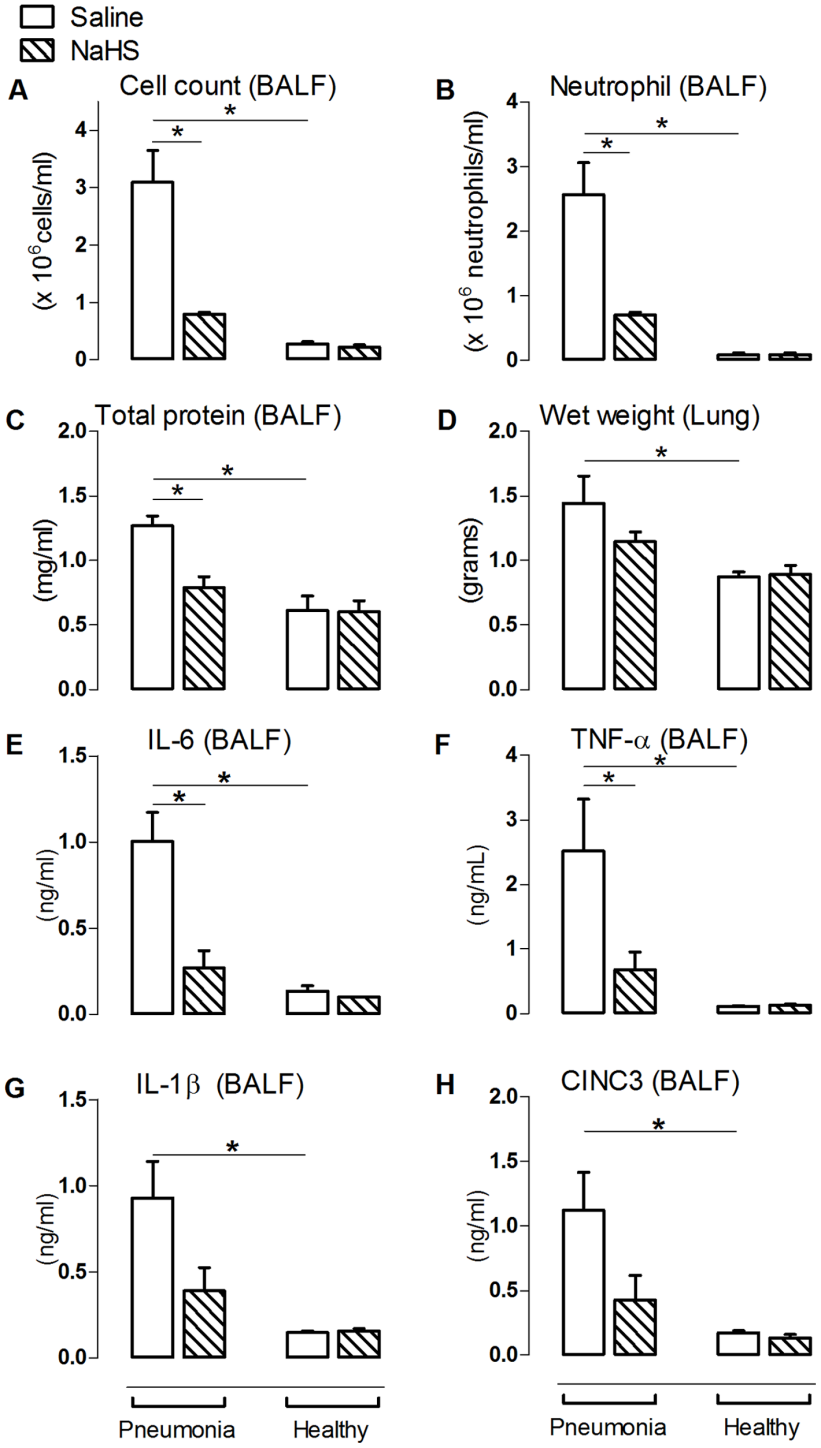
NaHS reduced lung injury parameters in pneumosepsis. The number of cells (A), neutrophil influx (B), total protein (C), interleukin (IL)–6 (E), TNF–α (F), IL–1β (G) and CINC3 (H) in animals infected with *S. pneumoniae* and healthy controls was determined in bronchoalveolar lavage fluid (BALF). The right lung lobes were used to determine lung wet weight (D). Mean ± SEM, (n = 6 in the pneumonia group infused with NaHS and n = 8 in the other experimental groups). *: p<0.05.

**Figure 2:**
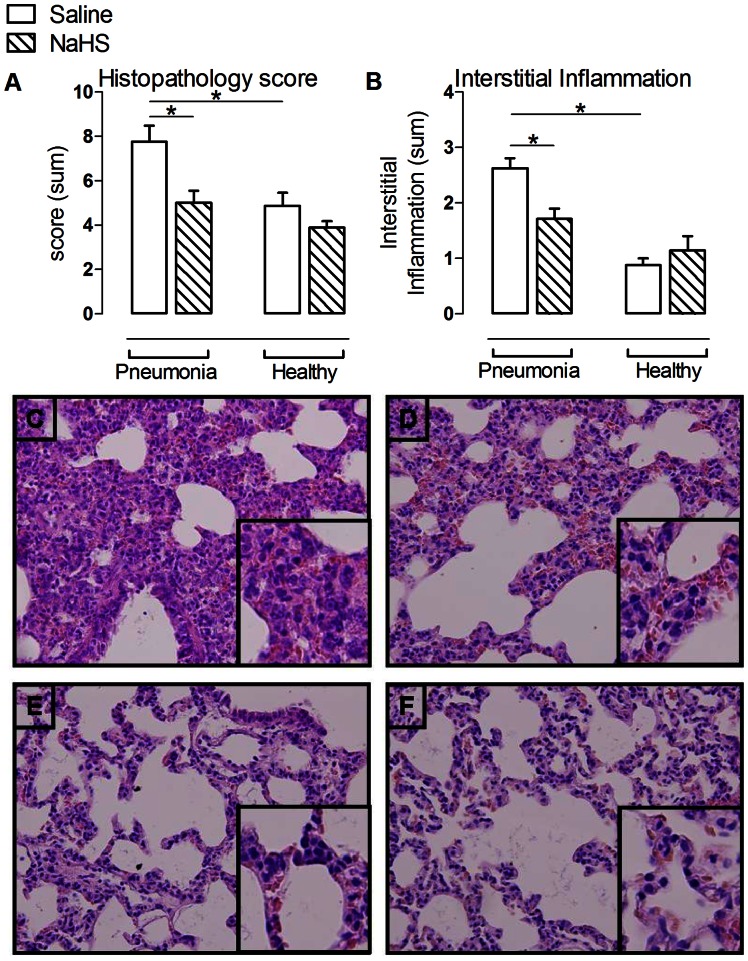
NaHS reduced histopathologic abnormalities and interstitial inflammation in pneumosepsis. Total histopathology score (A) and interstitial inflammation (B) with representative photographs of hematoxylin and eosin stained lung tissue sections (magnification×20) of animals infected with *S. pneumoniae* infused with saline (C) or NaHS (D) and healthy controls (E+F). (n = 6 in the pneumonia group infused with NaHS and n = 8 in the other experimental groups). Mean ± SEM, *:p<0.05. Inserts represents interstitial inflammation.

Pneumonia increased bronchoalveolar lavage fluid (BALF) protein content and lung wet weight compared to non-infected controls (p<0.05, figure 1C+D), accompanied by an enhanced pulmonary cell- and neutrophil influx (p<0.05, figure 1B), increased BALF levels of IL-1β, IL-6, TNF-α and CINC3 (p<0.05 for all, figure 1E–H) in addition to lung histopathological abnormalities (p<0.05, figure 2). Pneumonia resulted in increased levels of AF (48.5±10.4 vs. 29.1±3.7 U/L, p<0.05) in the BALF compared to non-infected controls, indicating cell damage. Pulmonary injury was accompanied by a decrease in arterial pO_2_ (p<0.05, table S1) and required higher respiratory frequencies and peak airway pressure to maintain normo-pH (p<0.05, table S1) compared to non-infected controls. Systemically, pneumonia caused modest inflammation, with increased plasma levels of IL-6, but not of TNF–α, IL-1β and CINC3 when compared to non-infected controls (p<0.05, figure S2). Pneumonia resulted in an increase in kidney wet weight and urine protein content (both p<0.05, table 1), indicating kidney vascular damage and permeability edema. Plasma levels of ALT, but not AST, were increased during pneumonia (p<0.05, table 1). No histopathological abnormalities were observed on liver and kidney H&E stained slides.

**Figure 3:**
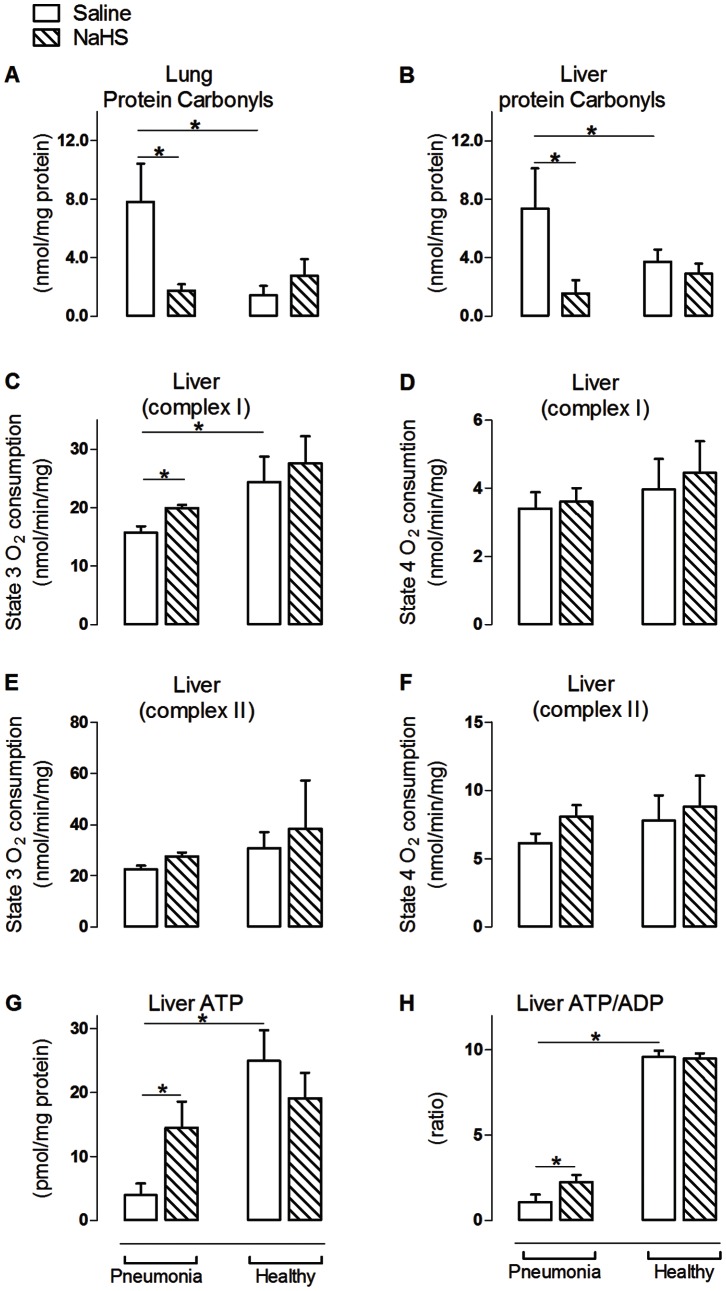
NaHS reduces oxidative damage and improved complex I mitochondrial respiration and ATP availability in pneumosepsis. Lung (A) and liver (B) protein carbonyls levels, with mitochondrial O_2_ consumption during oxidative phosphorylation of glutamate and malate (C+D), mitochondrial O_2_ consumption during oxidative phosphorylation of succinate (E+F) and concentrations of ATP (G) and ATP/ADP ratio (H) in animal infected with *S. pneumoniae* and healthy controls infused with saline or NaHS. Mitochondrial O_2_ consumption was recorded during state 3 and state 4 respirations in liver. Mean ± SEM, (n = 6 in the pneumonia group infused with NaHS and n = 8 in the other experimental groups). *p<0.05.

**Figure 4:**
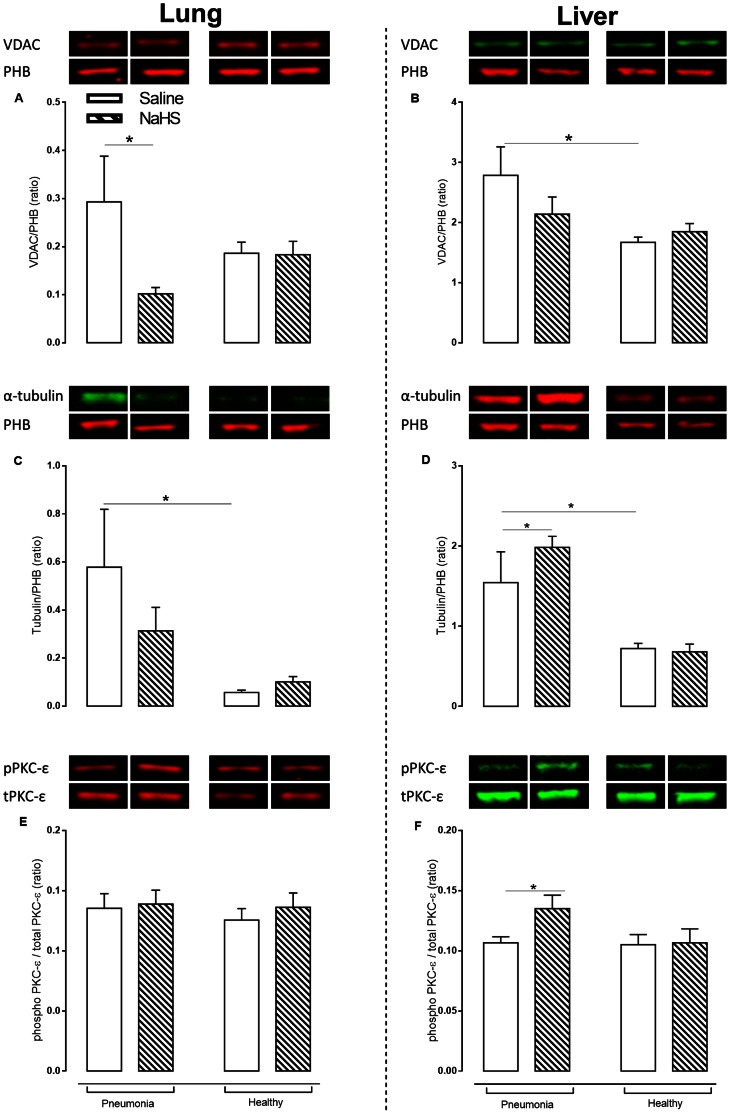
NaHS increased protein level of α-tubulin and protein kinase C (PKC)-ε as regulators of voltage dependent anion channel (VDAC) in pneumosepsis. Protein levels of VDAC (A+B), α-tubulin (C+D) and PKC-ε (E+F) on two separate immunoblots in lung and in liver freezed clamp biopsies of animals infected with *S. pneumoniae* or healthy controls infused with saline or NaHS. Data represent mean ± SEM from 6 animals per group. VDAC and α-tubulin are given as a ratio of prohibitin (PHB) expression. Phosphorylated PKC-ε is given as a ratio of total PKC-ε. Data as mean ± SEM, *: p<0.05.

**Figure 5:**
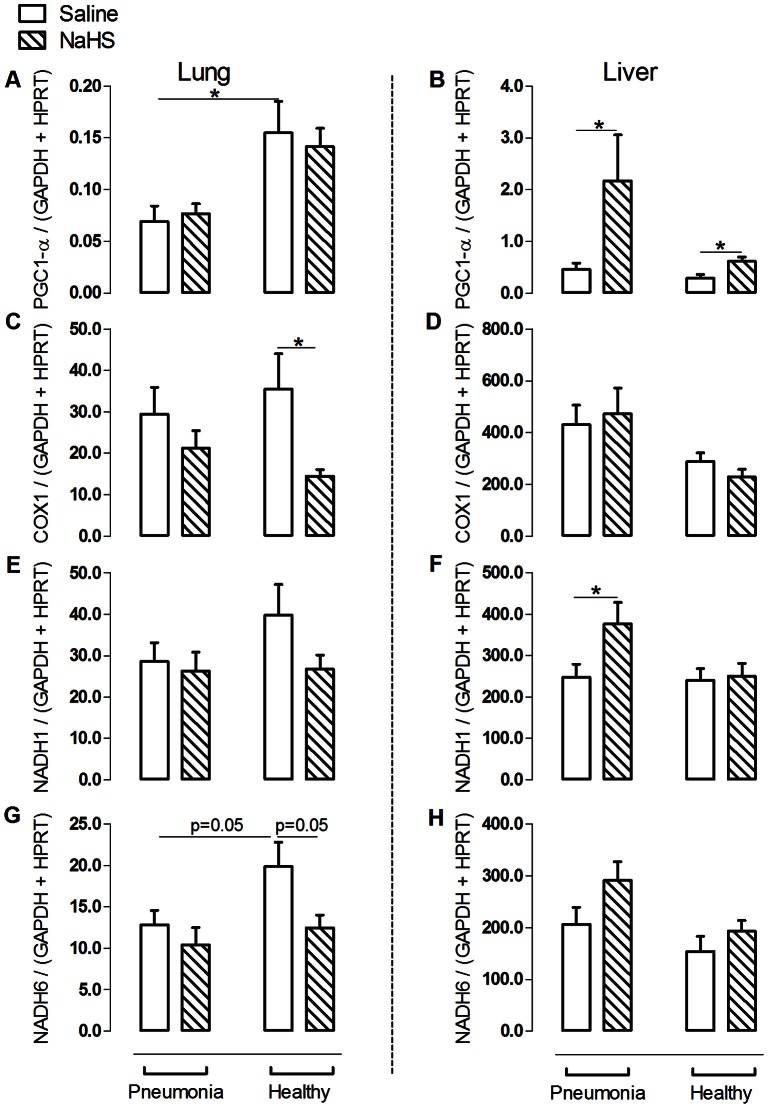
NaHS increased mitochondrial biogenesis by up regulation of expression of PGC-1α and mitochondrial respiratory subunits in the liver. The expression of peroxisome proliferator–activated receptor gamma (PGC)-1α (A+B), COX1 (C+D), NADH1 (E+F) and NADH6 (G+H) at mRNA level in lung and liver in animals infected with *S. pneumoniae* or healthy controls infused with saline or NaHS. Data represent mean ± SEM from n = 6 in the pneumonia group infused with NaHS and n = 8 in the other experimental groups. Data as mean ± SEM, *: p<0.05.

Pneumosepsis-induced inflammatory responses and organ injury were accompanied by oxidative damage, reflected in higher protein carbonyl levels in both organs (p<0.05, figure 3A+B). Mitochondrial oxygen consumption when challenged with substrate for complex I was markedly decreased during pneumosepsis compared to non-infected controls (p<0.05, figure 3C+D), with a concomitant decrease in ATP and ATP/ADP ratios (p<0.05, figure 3G+H). Respiration when challenged with substrate for complex II was unchanged in sepsis (figure 3E+F). VDAC expression on the other hand was increased during pneumosepsis, reflecting a low bio-energetic status and enhanced ATP demand (p<0.05, figure 4B). Also α-tubulin was increased compared to non-infected controls (p<0.05, figure 4C+D). Low bio–energetic status was associated with decreased PGC1–α expression, a metabolism regulator [10] with concomitant low expression of mitochondrial respiratory subunit NADH6 in the lung (p<0.05, figure 5A+G) as compared to non-infected controls. Expressions of other subunits were not affected.

### NaHS did not Alter Bacterial Outgrowth of *S. pneumoniae*

NaHS did not influence bacterial outgrowth in BALF and lung homogenates compared to saline controls, nor did it influence bacterial outgrowth in distant organs (figure S1). NaHS tended to decrease the number of positive blood cultures (38% vs. 13% in saline controls, p = 0.13).

### NaHS Reduced Heart Rate, Body Temperature and Improved Gas Exchange

In line with our previous experiments [18], infusion of NaHS reduced heart rate and body temperature compared to saline controls, both in pneumonia and in healthy rats, without an effect on mean arterial pressure (all p<0.05, table 1). NaHS allowed for a reduction in respiratory rates compared to saline controls while maintaining normo-pH, with 20% reduction in infected and 26% in non-infected animals (p<0.05, table S1). NaHS prevented a fall in arterial pO_2_ in pneumonia and increased oxygenation in the healthy animals (p<0.05, table S1), but did not influence peak airway pressure.

### NaHS Reduced Local and Systemic Inflammation and Ameliorated Kidney Function in Pneumosepsis

NaHS reduced BALF protein concentration compared to saline controls (p<0.05, figure 1C), but did not reduce pulmonary edema. Also, NaHS reduced pulmonary cell counts and neutrophils influx as well as BALF levels of IL-6 and TNF-α (all p<0.05, figure 1E+F). NaHS non-significantly decreased BALF levels of IL-1β and CINC3 compared to saline controls (p = 0.08 and 0.18 respectively, figure 1G+H). Inhibition of inflammation by NaHS was also reflected in the lung histopathology score, in particular interstitial inflammation (p<0.05, figure 2). NaHS did not affect AF concentration (48.5±10.4 vs. 29.1±3.7 U/L) compared to saline controls. NaHS decreased systemic levels of IL-6 (p<0.05, figure S2A), but levels of TNF-α, IL-1β and CINC3 were not altered. NaHS infusion reduced kidney wet weight and protein loss in the urine (both p<0.05, table 1), together with an improved glomerular filtration rate and reduced plasma creatinine levels compared to saline controls (p<0.05, table 1), while cumulative fluid infusion nor diuresis differed. Liver enzymes were reduced by NaHS, which did not reach statistical significance due to a wide range (p = ns, table 1).

### NaHS Prevented a Fall in Bio-energetic Status by Maintaining Mitochondrial Function and by Upregulation of Mitochondrial Biogenesis

Infusion of NaHS prevented the observed increase in levels of protein carbonyls (p<0.05, figure 3A+B) in pneumonia compared to non-infected controls. In line with reduced oxidative stress, NaHS prevented the fall in active oxidative phosphorylation observed during pneumonia when challenged with substrate for complex I (p<0.05, figure 3C), together with an increase in ATP concentration and ATP/ADP ratio (p<0.05, figure 3G+H). Infusion of NaHS had no effect on the expression of VDAC, but resulted in upregulation of α-tubulin and the fraction of phosphorylated PKCε as regulators of VDAC in liver mitochondria (p<0.05, figure 4C–F). Effects of NaHS infusion on parameters of mitochondrial function differed between the lung, which was the primary diseased site, and the liver, a sentinel organ in regulating metabolism. In the lung, the effect of NaHS on regulators of VDAC was not observed. Furthermore, we found that infusion of NaHS reduced levels of COX1 and tended to reduce levels of NADH1 in BALF, suggesting less leakage of damaged lung mitochondria (figure S3 B+D). Circulating levels of COX1, NADH1 and NADH6 were decreased in a non-significant fashion by NaHS.

Besides improved mitochondrial function, NaHS infusion increased expression of liver PGC-1α, which is the main mitochondrial transcription factor (p<0.05, figure 5). NaHS also upregulated PGC1-α in healthy controls, indicating an ubiquitous effect of NaHS. In line with these results, hepatic NADH1 expression increased and NADH6 expression tended to increase after NaHS infusion (figure 5F+H). Both are subunits of mitochondrial respiratory complexes. Taken together, these results may point to an enhanced mitochondrial biogenesis. Of note, we again found differences between the lung and liver. Upregulation of factors involved in mitochondrial biogenesis was noted in the liver, but not in the (diseased) lung.

## Discussion

In this model of pneumococcal pneumosepsis, infusion of H_2_S-donor NaHS reduced organ damage, associated with maintained mitochondrial integrity by reducing oxidative damage as well as with increased mitochondrial biogenesis, which may both have contributed to a reversal of inhibition of mitochondrial oxygen consumption and increased ATP bio-availability.

NaHS reduced lung injury by inhibiting inflammatory processes, in accordance with previous findings in non-infectious models of acute lung injury caused by smoke inhalation [22] or induced by mechanical ventilation [18]. NaHS reduced pulmonary neutrophil influx and protein leakage in the alveolar compartment during pneumonia. Preservation of the endothelial barrier may have been a mechanism of the observed decrease in distant organ injury in this study, as biomarkers of endothelial damage have been found in blood of patients with pneumonia, contributing to multiple organ failure [23].

Acute kidney injury is a common complication of pneumosepsis, with an important impact on outcome [24]. In our model, pneumonia resulted in edema of the kidney, with urinary protein loss and deterioration of kidney function. Infusion of NaHS reversed the fall in the glomerular filtration rate and kidney function both in pneumonia as well as in healthy animals, suggesting that NaHS improved perfusion of the kidney. Decrease of protein leakage may presumably be due to anti-inflammatory effects of NaHS, thereby preserving the endothelial barrier in the glomeruli, comparable to effects in the pulmonary compartment. NaHS tended to decrease liver cell damage, as reflected in decreased levels of both AST and ALT. We presume that significance was not reached due to a large variation in the levels of the liver enzymes, which would possibly be overcome with larger group size.

Under normal conditions, the electron transport chain complexes in the inner mitochondrial membrane are involved in formation of ATP. In sepsis, complex I and II are susceptible to damage, [25] which may lead to decreased ATP concentrations at cellular levels. We found that in pneumonia, mitochondrial respiration was decreased when challenged with substrate for complex I, accompanied by reduced ATP and ATP/ADP ratios, while respiration with substrate for complex II was unchanged. This is in line with findings in human septic shock [5]. Low ATP levels in combination with a high expression of VDAC and impaired oxygen consumption during pneumonia, is suggestive of an increased need for ATP in the presence of mitochondrial damage, presumably due to increased levels of pro-inflammatory cytokines and reactive oxygen species, as found in this study.

In sepsis, reactive oxygen species not only damage mitochondrial respiratory complexes, but also other vital proteins [26], thereby forming protein carbonyls [27]. We found a reduction of protein carbonyls after NaHS infusion, which may have contributed to reversal of the fall in mitochondrial respiration, together with increased ATP levels. With respect to the mechanism of improved mitochondrial respiration, we found that NaHS increased the expression of proteins in the mitochondrial fraction which induce closure of VDAC, including α-tubulin [12] and PKCε [28]. VDAC is considered an important regulator of mitochondrial respiration [29]. We suggest that NaHS preserves mitochondrial function during sepsis by increasing expression of mitochondrial α-tubulin and PKCε, thereby closing VDAC and stabilizing the mitochondrial membrane [30], resulting in preservation of oxidative phosphorylation. However, as we did not measure activity of other ATP-ADP transporters, definite statements about the mechanisms of improved ATP bioavailability cannot be made.

Another indication that mitochondrial damage was limited by NaHS is the finding that levels of mtDNA in the bronchoalveolar lavage fluid were reduced, suggesting less leakage of mitochondria [31]. As circulating or shed mtDNA invokes an inflammatory response in sepsis and trauma [31], a reduction in damaged mitochondria may have contributed to the protective effect of NaHS in this study. Although systemic mtDNA levels were non-significantly reduced after NaHS, we found a trend in decrease for all subunits, suggestive of a lack of power to demonstrate a significant effect.

Besides maintaining oxidative phosphorylation, we also found evidence for an improved mitochondrial biogenesis after NaHS infusion, as concluded from the upregulated expression of PGC1-α and transcription factors coding for respiratory complexes in pneumonia. Similarly to the effects of NaHS on mitochondria, other gas molecules like carbon monoxide and nitric oxide are able to enhance mitochondrial biogenesis [32].

The effects of NaHS on regulators of mitochondrial function were most evident in the liver, but not in the lung. The liver is a sentinel organ in regulating metabolism [32], [33], whereas the lung is not. Also, the lungs in this study were considerably damaged by pneumonia, which may underlie a lack of effect on mitochondrial biogenesis.

We previously reported that NaHS reversibly reduced metabolism in anesthetized rats [18], akin to the induction of a suspended animation like-state [17]. It has been stated that reducing metabolism with concomitant hypothermia may render the host more susceptible to infection [34], [35], although infection risk differs among studies [34]–[36]. We did not find enhanced dissemination of bacteria during the hypothermic period. In contrast, there was a tendency to local containment of bacteria. This may result from the improved endothelial barrier function by NaHS, thereby inhibiting dissemination. However, an important question remains whether bacteria restart replicating after cessation of NaHS.

It may be argued that observed protective effects are attributable to hypothermia induced by NaHS. Although mild hypothermia reduces inflammation [37]–[39], detrimental effects of deep hypothermia have been reported in experimental settings [40], [41] and in humans [42]. In accordance, induction of deep hypothermia that paralleled effects of NaHS either worsened lung inflammation or had no effect in a rat model of ventilator induced lung injury [18], [43] or shock [44], [45]. Thereby, we hypothesize that reducing metabolism by inhibiting mitochondrial respiration by NaHS while not allowing to reduce body temperature may be toxic.

A limitation of this study may be that mitochondrial respiration was measured at 37°C, which does not represent the *in vivo* situation in NaHS infused animals. However, as the technique was the same in all groups, limitations pertaining to the measurement cannot explain differences between NaHS and controls. Finally, we did not measure H_2_S concentrations in tissue, which is a limitation to our study. However, NaHS infusion may be a more feasible treatment strategy than H_2_S gas administration. Therefore, we consider results of this study relevant for future studies evaluating the use of NaHS in sepsis.

### Conclusion

In this model of severe pneumosepsis, NaHS reduced local and distant organ injury, associated with maintaining mitochondrial function as well as with improving mitochondrial biogenesis. Interventions aimed at stabilizing bio-energetic status may be a therapeutic approach to reduce organ failure in pneumosepsis.

## Supporting Information

Figure S1 NaHS did not reduce bacterial outgrowth. The number of colonizing forming units (CFU) in bronchoalveolar lavage fluid (BALF) (A), lung (B), liver (C) and spleen (D) homogenates in animals infected with *S. pneumoniae* were determined by 10 fold dilutions on blood agar plates (n = 8 in pneumonia group and n = 6 in the NaHS group). Horizontal line indicates the mean (log scale).
(TIF)

Figure S2 NaHS reduced systemic levels of interleukin (IL)–6 in pneumonia. The concentrations of IL–6 (A), tumor necrosis factor (TNF)–α (B), IL–1β (C) and CINC3 (D) in plasma of animals infected with *S. pneumoniae* and healthy controls infused with saline or H_2_S. (n = 6 in the pneumonia group infused with H_2_S and n = 8 in the other experimental groups). Mean ± SEM. *: p<0.05.
(TIF)

Figure S3 NaHS maintained mitochondrial integrity reflected by low levels of mitochondrial DNA in bronchoalveolar lavage fluids (BALF). The expression of COX1 (A+B), NADH1 (C+D) and NADH6 (E+F) in plasma and in BALF in animals infected with *S. pneumoniae* or healthy controls infused with saline or H_2_S. Data represent mean ± SEM from n = 6 in the pneumonia group infused with NaHS and n = 8 in the other experimental groups.
(TIF)

Table S1 (DOC)

Methods S1 (DOCX)
