# Supplementary figures and images for: Hydrogen Sulfide Donor NaHS Reduces Organ Injury in a Rat Model of Pneumococcal Pneumosepsis, Associated with Improved Bio-Energetic Status
Source: PLoS One. 2013 May 23;8(5):e63497. doi: 10.1371/journal.pone.0063497 (PMC3662774; doi:10.1371/journal.pone.0063497)

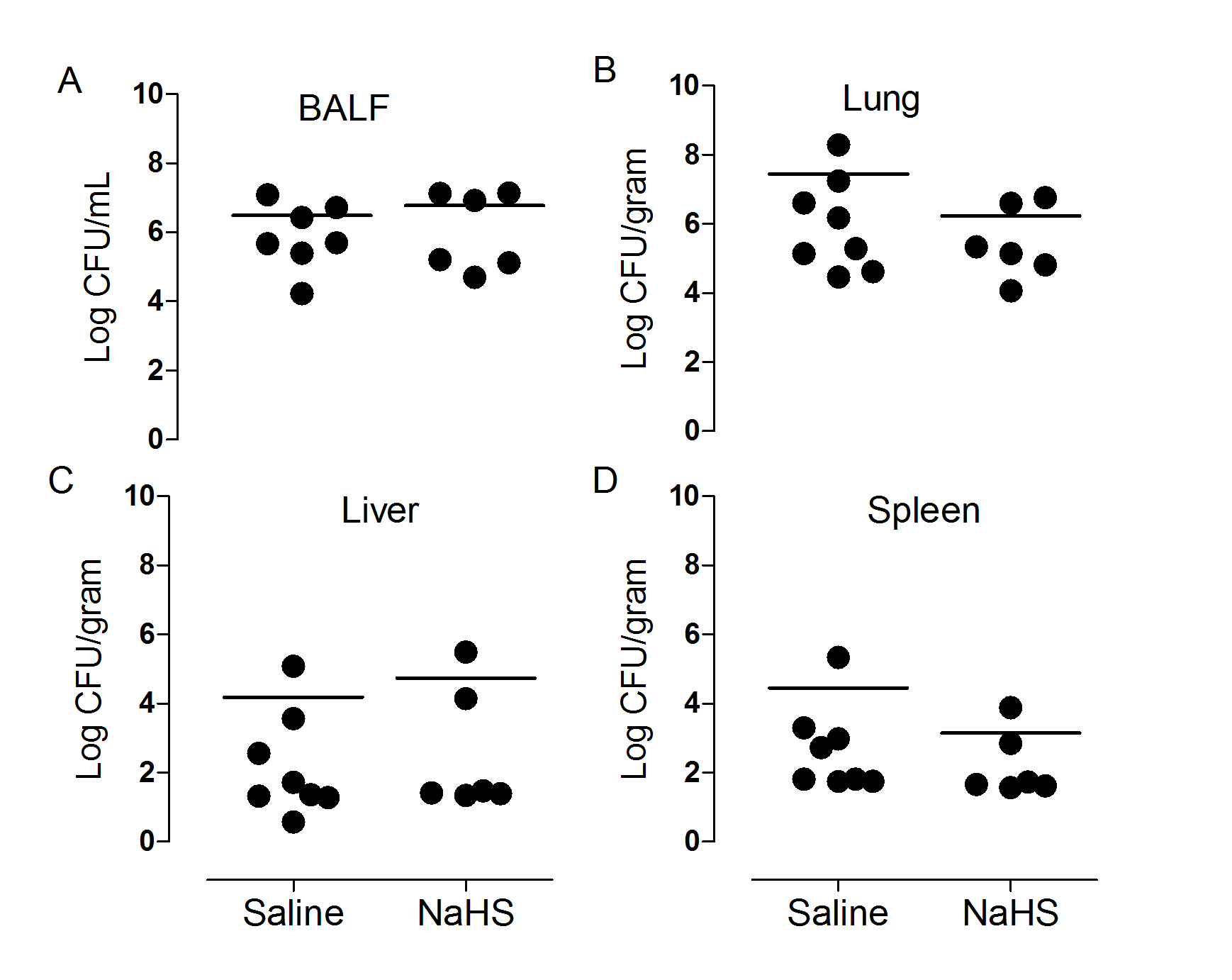

Supplement: Figure S1 — NaHS did not reduce bacterial outgrowth. The number of colonizing forming units (CFU) in bronchoalveolar lavage fluid (BALF) (A), lung (B), liver (C) and spleen (D) homogenates in animals infected with S. pneumoniae were determined by 10 fold dilutions on blood agar plates (n = 8 in pneumonia group and n = 6 in the NaHS group). Horizontal line indicates the mean (log scale). (TIF) [file pone.0063497.s001.tif]

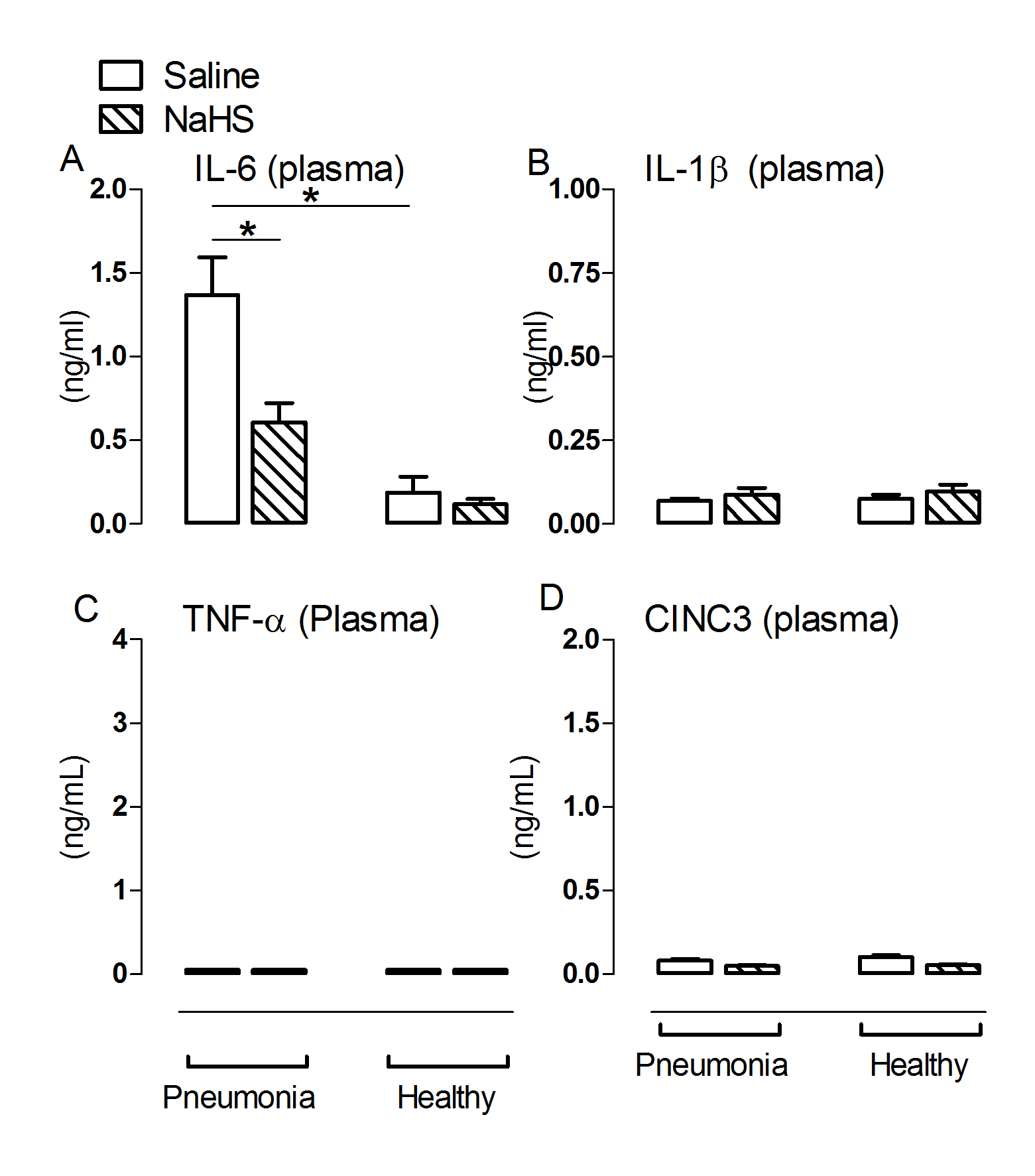

Supplement: Figure S2 — NaHS reduced systemic levels of interleukin (IL)–6 in pneumonia. The concentrations of IL–6 (A), tumor necrosis factor (TNF)–α (B), IL–1β (C) and CINC3 (D) in plasma of animals infected with S. pneumoniae and healthy controls infused with saline or H2S. (n = 6 in the pneumonia group infused with H2S and n = 8 in the other experimental groups). Mean ± SEM. *: p<0.05. (TIF) [file pone.0063497.s002.tif]

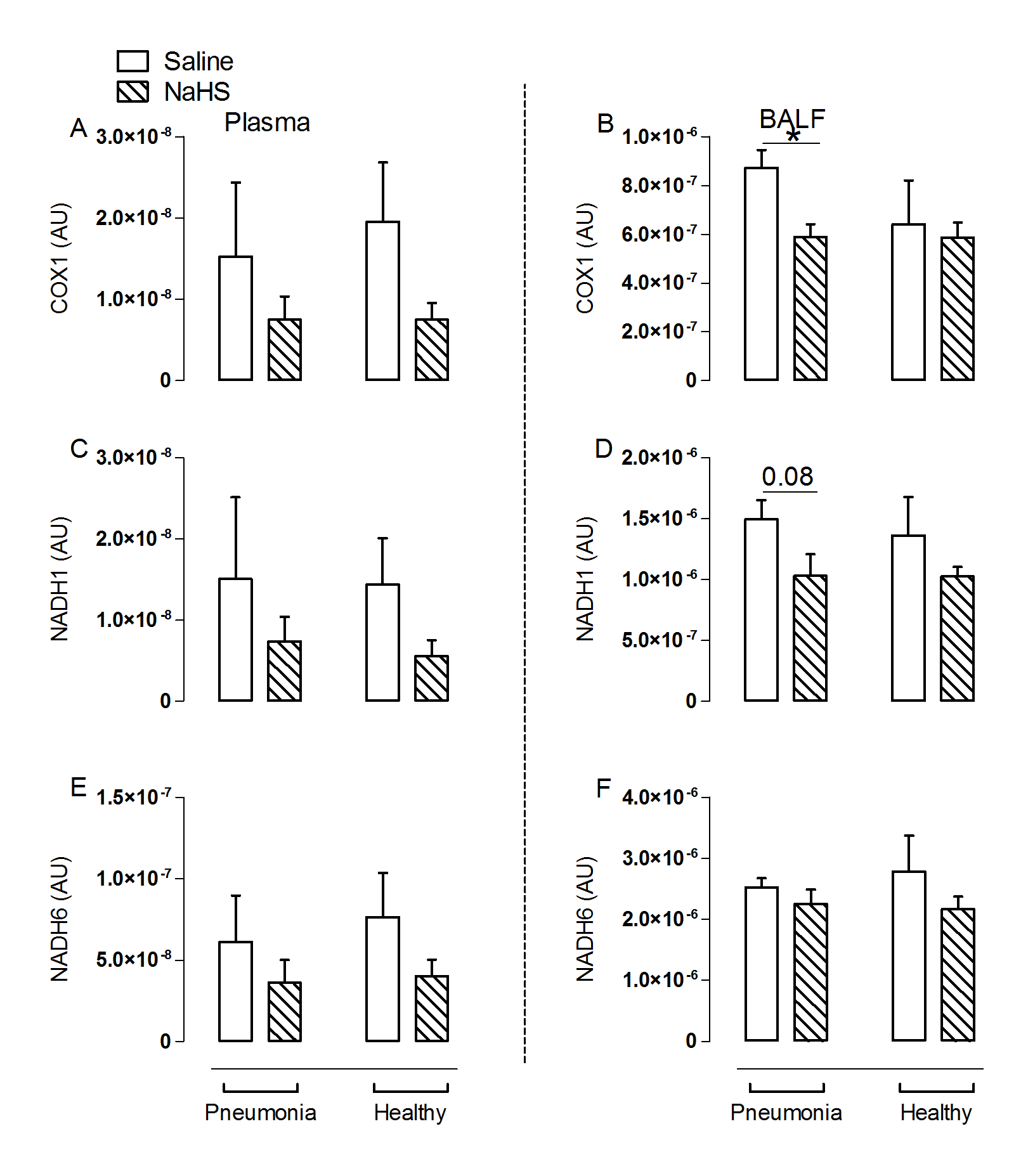

Supplement: Figure S3 — NaHS maintained mitochondrial integrity reflected by low levels of mitochondrial DNA in bronchoalveolar lavage fluids (BALF). The expression of COX1 (A+B), NADH1 (C+D) and NADH6 (E+F) in plasma and in BALF in animals infected with S. pneumoniae or healthy controls infused with saline or H2S. Data represent mean ± SEM from n = 6 in the pneumonia group infused with NaHS and n = 8 in the other experimental groups. (TIF) [file pone.0063497.s003.tif]
