# Supplementary material for: Hydrogen Sulfide Donor NaHS Reduces Organ Injury in a Rat Model of Pneumococcal Pneumosepsis, Associated with Improved Bio-Energetic Status
Source: PLoS One. 2013 May 23;8(5):e63497. doi: 10.1371/journal.pone.0063497 (PMC3662774; doi:10.1371/journal.pone.0063497)
Supplement: Table S1 — (DOC) [file pone.0063497.s004.doc]

**Table S1**

| **Table S1:** Arterial blood gas analysis and respiratory parameters at baseline (t=0) and after 4 hours of NaHS or saline infusion in rats with pneumosepsis and in healthy controls. | | | | | |
| --- | --- | --- | --- | --- | --- |
|  | | **Pneumonia** | | **Healthy** | |
|  | **Time (hour)** | **Saline** | **NaHS** | **Saline** | **NaHS** |
| pH | T = 0 | 7.39±0.07 | 7.49±0.06 | 7.37±0.07 | 7.47±0.03 |
|  | T = 4 | 7.35±0.07 | 7.46±0.06 | 7.36±0.07 | 7.45±0.11 |
| PaCO_2_ (kPa) | T = 0 | 5.2±0.5 | 4.2±0.4 | 5.6±1.0 | 3.8±0.8 |
|  | T = 4 | 4.9±0.9 | 3.7±0.8* | 5.0±0.9 | 3.7±0.8 |
| PaO_2_ (kPa) | T = 0 | 29±5 | 30±6 | 31±2 | 34±2 |
|  | T = 4 | 24±5† | 41±6* | 31±4 | 42±4^#^ |
| HCO_3_^-^ (mmol/L) | T = 0 | 23±2 | 24±2 | 22±5 | 20±3 |
|  | T = 4 | 20±5 | 19±3 | 21±3 | 20±2 |
| Base excess | T = 0 | -1.3±3.1 | 1.1±2.4 | -1.6±1.4 | -2.0±2.0 |
|  | T = 4 | -4.9±5 | -3.4±2.0 | -4.2±3.0 | -3±3 |
| Respiratory rate (breaths/min) | T = 0 | 45±0† | 45±0 | 35±0 | 35±0 |
|  | T = 4 | 50±5 | 40±5* | 42±6 | 31±4^#^ |
| Peak pressure (cmH_2_O) | T = 0 | 18±0† | 19±1 | 16±0 | 17±1 |
|  | T = 4 | 18±0† | 19±1 | 17±1 | 18±1 |
| Data are means ± SD. *: Pneumonia + saline vs. pneumonia + NaHS, # healthy + saline vs. healthy + NaHS, †: pneumonia vs. healthy, p<0.05.  p<0.05. | | | | | |
