# Supplementary material for: Hydrogen Sulfide Donor NaHS Reduces Organ Injury in a Rat Model of Pneumococcal Pneumosepsis, Associated with Improved Bio-Energetic Status
Source: PLoS One. 2013 May 23;8(5):e63497. doi: 10.1371/journal.pone.0063497 (PMC3662774; doi:10.1371/journal.pone.0063497)
Supplement: Methods S1 — (DOCX) [file pone.0063497.s005.docx]

**Title:** Hydrogen sulfide donor NaHS reduces organ injury in pneumosepsis, associated with improved bio-energetic status.

**Methods S1**

*Mechanical ventilation*

Ventilator settings were determined in pilot experiments aiming at normo-pH (7.35-7.45) and tidal volumes of ~7.5 ml/kg [1]. Rats with pneumonia were ventilated in a pressure controlled mode with 14 cmH_2_O peak inspiratory pressure (PIP), 5 cm H_2_O positive end expiratory pressure (PEEP). Non-infected rats were ventilated with 12 cmH_2_O PIP and 5 cmH_2_O PEEP. FiO_2_ was set at 50%, inspiration to expiration ratio at 1:2, recruitment maneuvers were applied every 60 minutes by increasing PIP to 25 cmH_2_O during 5 breaths. In pneumonia respiratory rate at baseline was 45 breaths/min and in the healthy controls 35 breaths/min. Respiratory rates were adjusted to maintain normo-pH in all groups.

*Assays in BALF, plasma and tissue*

*Freezeclamp and mitochondrial isolation*

Part of the liver was removed by modified freeze clamping and placed in liquid nitrogen for ATP and adenosine diphosphate (ADP) measurements and part was placed in isolation buffer. To minimize ATP degradation and for mitochondrial viability, these steps were performed before exsanguination. For mitochondrial isolation, part of the liver was minced in isolation buffer (200 mmol/L mannitol, 50 mmol/L sucrose, 5 mmol/L KH_2_PO4, 5 mmol/L 3-(n-morpholino) propanesulfonic acid (MOPS), 1 mmol/L Ethylene glycol−bis(2−aminoethylether)−N,N,N′,N′−tetraacetic acid (EGTA), 0.1% bovine serum albumin), homogenized and centrifuged at 3220 x g for 10 min. Supernatant was removed, and the pellet was resuspended in 25 mL isolation buffer and centrifuged at 800 x g for 10 min. The supernatant was centrifuged at 3220 x g for 10 min. The final pellet was suspended in isolation buffer. Protein content was determined by the Bradford method (Bio–Rad, München, Germany). The isolation steps were conducted at 4ºC.

*Mitochondrial oxygen consumption*

Mitochondrial oxygen consumption was measured polarographically at 37 °C using a respiratory system (System S 200A, Strathkelvin Instruments, Glasgow, Scotland). Mitochondria (2 mg protein/ml) were suspended in respiration buffer (130 mmol/L KCl, 5 mmol/L K_2_HPO_4_, 20 mmol/L MOPS, 2.5 mmol/L EGTA, 1 μmol/L Na_4_P_2_O_7_, 0.1% BSA, pH adjusted). Shortly after a stable signal was reached, mitochondrial respiration was initiated using glutamate (20mM, Aldrich, Steinheim, Germany) with malate (20mM, Aldrich, Steinheim, Germany). Exactly 60 s later, state 3 respiration was initiated by 200 μmol/L ADP injected into the respiration chamber. Respiration rates were recorded under state 3 conditions for approximately 4 minutes or after complete phosphorylation of ADP to ATP (state 4 respiration).

*ATP and ADP determination*

For nucleotide extraction, approximately 40-100 mg frozen tissue was grinded in liquid nitrogen using a ceramic mortar. Right before the liquid nitrogen has evaporated, the semi-viscous tissue powder was transferred to an eppendorf tube containing 200 µL of 0.4 M HCIO_4_ and placed on ice for 10 minutes. After centrifugation (10.000 x g for 10 minutes at 4ºC), 150 µL of the supernatant was transferred to an empty Eppendorf tube and neutralized to pH 7.5-8.5 with 5 M K_2_CO_3_. The remainder of the supernatant was removed and 1000 µL of 0.2 M NaOH was added to the pelleted cell fragments, which was used for protein determination (BCA assay, Pierce, Etten-Leur, the Netherlands). The supernatants containing the nucleotides, including ATP and ADP, were stored at -80 until analysis by high performance liquid chromatography (HPLC).

*Nucleotide analysis by HPLC*

Nucleotide profiles were determined by ion-exchange HPLC, using a Whatman Partisphere SAX 4.6 × 125 mm column (5-*µ*m particles) in combination with a Whatman 10 × 2.5 mm AX guard column (Clifton, NJ). The buffers used were 9 mM NH_4_H_2_PO_4_, pH 3,5 (buffer A) and 325 mM NH_4_H_2_PO_4_, 500 mM KCL, pH 4.4 (buffer B). Nucleotides were eluted with a gradient from 100% buffer A to 90% buffer B in a total run time of 60 min, at a flow-rate of 1mL/min [2]. The concentration of ATP and ADP were corrected for protein concentrations.

*Mitochondrial fraction*

Part of the liver freeze clamp biopsy and snapfrozen lung part was crushed in a pre-cooled mortar and quickly carried over to lysis buffer (Tris base, EGTA, NaF and Na_3_VO_4_ (as phosphatase inhibitors) containing protease inhibitor mix (aprotinin, leupeptin and pepstatin) and DTT and homogenized on ice. The liver homogenates were centrifugated at 1000 x g, 4 ºC for 10 min. To obtain the mitochondrial fraction, the supernatant was centrifuged at 10,000 x g, 4 ºC for 15 min. Again, the supernatant was centrifugated at 16,000 x g, 4 ºC for 15 min to obtain the cytosolic fraction.

*Western Blot analysis*

Total protein concentration was determined by the Lowry method. Subsequently, proteins were separated on a 4%-12% gradient gel (Criterion^TM^ XT precast gel, Bio-Rad, the Netherlands) and transferred to a polyvinylidene fluoride membrane (Immobilon FL, Millipore, the Netherlands) by tank blotting. To prevent unspecific binding of the antibody, membranes were incubated with Odyssey Blocking Buffer (LI-COR, Westburg, Leusden, the Netherlands) with 0.1 % Tween (TBS-T) for 1 hour. Subsequently, the membrane was incubated over night at 4°C with the respective primary antibody anti-α-tubulin (Sigma Aldrich, St. Lous, MO), anti-VDAC (Calbiochem, Darmstadt, Germany), anti-phosphor PKCε, anti-total PKCε (both Biomed, Huisen, the Netherlands) or anti-prohibitin 1 (PHB) (Cell Signal, Danvers, United States) diluted in 0.1% Tween Odyssey buffer. After washing in fresh, cold TBS-T 3x 5 min, the membrane was subjected to the appropriate secondary antibody for 1 hour at room temperature. Immuno-reactive bands on the membrane were visualized by the two channel laser system of the Odyssey system. The blots were quantified using the Odysses IR Manager®. Equal loading of the protein to the SDS Page gel was ensured by Coomassie blue staining (Bio-Rad, Veenendaal, the Netherlands) and Ponceau staining (Sigma Aldrich).

*Lung, liver, plasma and BALF mRNA isolation*

Total RNA was extracted with Tripure (Roche diagnostics BV, Almere, Netherlands) from approximately 30 mg lung and liver tissue according to manufacturers’ instruction. Total DNA was isolated in 200µL plasma and BALF with Qiamp DNA kitt (Qiagen, Venlo, Netherlands). RNA and DNA samples were quantified by spectrophotometry and stored at -80. RNA was converted to cDNA by using oligo dT as primers. Rat, COX1 (forward: 5’ATGACCCACCAATCACATGC3’ reverse: 5’ATCACATGGCTAGGCCGGAG3’), NADH1 (forward: 5’ATACCCATGGCCAACCTCCT3’ reverse: 5’GGGCCTTTGCGTAGTTGTAT3’), NADH6 (forward: 5’CTCACATGACAAAAACTAGCCCCCA3’ reverse 5’TCCACCTCAACTGCCTGCTATGA3’), and PGC-1α (forward: 5’CAGAACCATGCAAACCACAC3’ reverse: 5’TTGTGGCTTTTGCTGTTGAC3’) was analyzed by reverse-transcription-polymerase chain reactions (RT-PCRs) using lightCycler®SYBR green I master mix (Roche, Mijdrecht, the Netherlands) and measured in a LightCycler 480 (Roche) apparatus using the following conditions: 5 min 95° C hot-start, followed by 40 cycles of amplification (95° C for 10 seconds, 60° C for 5 seconds, 72° C for 15 seconds). Gene expression is presented as a ratio of the mean expression of the housekeeping genes HRPT (forward: 5’CCATCTTCCAGGAGCGAGAT3’, reverse: 5’CTCCATGGTGGTGAAGACG3’) and GAPDH (forward: 5’CCAGACAAGTTTGTTGTTGGA3’, reverse: 5’GCTTTTCCACTTTCGCTGAT3’).

**Supplemetal references**

1. Aslami H, Heinen A, Roelofs JJ, Zuurbier CJ, Schultz MJ, et all (2010) Suspended animation inducer hydrogen sulfide is protective in an in vivo model of ventilator-induced lung injury. Intensive Care Med 36: 1946-1952. 10.1007/s00134-010-2022-2 [doi].

2. Bierau J, van Gennip AH, Helleman J, van Kuilenburg AB (2001) The cytostatic- and differentiation-inducing effects of cyclopentenyl cytosine on neuroblastoma cell lines. Biochem Pharmacol 62: 1099-1105. S0006-2952(01)00756-0 [pii].
